# Early microglial and astrocyte reactivity in preclinical Alzheimer's disease

**DOI:** 10.1002/alz.70502

**Published:** 2025-08-01

**Authors:** Marta Fernández‐Matarrubia, Andrea Valera‐Barrero, Mónica Renuncio‐García, Marcos Aguilella, Carmen Lage, Sara López‐García, J Gonzalo Ocejo‐Vinyals, Francisco Martínez‐Dubarbie, Guglielmo Di Molfetta, Ana Pozueta‐Cantudo, María García‐Martínez, Andrea Corrales‐Pardo, María Bravo, Marcos López‐Hoyos, Juan Irure‐Ventura, Kaj Blennow, Nicholas J Ashton, Henrik Zetterberg, Pascual Sánchez‐Juan, Eloy Rodríguez‐Rodríguez

**Affiliations:** ^1^ Neurology Service Marqués de Valdecilla University Hospital Santander Cantabria Spain; ^2^ Institute for Research Marqués de Valdecilla (IDIVAL) Santander Cantabria Spain; ^3^ CIBERNED, Network Center for Biomedical Research in Neurodegenerative Diseases National Institute of Health Carlos III Madrid Spain; ^4^ Immunology Department Marqués de Valdecilla University Hospital Santander Spain; ^5^ Atlantic Fellow for Equity in Brain health, Global Brain Health Institute University of California San Francisco USA; ^6^ Department of Psychiatry and Neurochemistry, Institute of Neuroscience and Physiology The Sahlgrenska Academy at the University of Gothenburg Gothenburg Sweden; ^7^ Molecular Biology Department University of Cantabria Santander Spain; ^8^ Clinical Neurochemistry Laboratory Sahlgrenska University Hospital Gothenburg Sweden; ^9^ Paris Brain Institute, ICM, Pitié‐Salpêtrière Hospital Sorbonne University Paris France; ^10^ Neurodegenerative Disorder Research Center, Division of Life Sciences and Medicine, and Department of Neurology, Institute on Aging and Brain Disorders University of Science and Technology of China and First Affiliated Hospital of USTC Hefei P.R. China; ^11^ Centre for Age‐Related Medicine Stavanger University Hospital Stavanger Norway; ^12^ Institute of Psychiatry, Psychology and Neuroscience, Maurice Wohl Clinical Neuroscience Institute King's College London London UK; ^13^ NIHR Biomedical Research Centre for Mental Health and Biomedical Research Unit for Dementia at South London and Maudsley NHS Foundation London UK; ^14^ Department of Neurodegenerative Disease UCL Institute of Neurology London UK; ^15^ UK Dementia Research Institute at UCL London UK; ^16^ Hong Kong Center for Neurodegenerative Diseases Hong Kong China; ^17^ Wisconsin Alzheimer's Disease Research Center, University of Wisconsin School of Medicine and Public Health University of Wisconsin‐Madison Madison Wisconsin USA; ^18^ Alzheimer's Centre Reina Sofia‐CIEN Foundation‐ISCIII Madrid Spain; ^19^ Deparment of Medicine and Psychiatry University of Cantabria Santander Spain

**Keywords:** astrocyte, biomarkers, chitinase‐3‐like protein 1 (YKL‐40), glial fibrillary acidic protein (GFAP), microglia, preclinical Alzheimer´s disease, S‐100 calcium‐binding protein beta (S100β), soluble triggering receptor expressed on myeloid cells 2 (sTREM2), structural equation modeling

## Abstract

**INTRODUCTION:**

The role of neuroinflammation in preclinical Alzheimer's disease (AD) remains unclear.

**METHODS:**

We assessed changes in microglial and astrocytic biomarkers in a well‐characterized cohort of 211 cognitively unimpaired individuals. Structural equation modeling was used to simultaneously assess all relationships among microglial and astrocytic responses and AD pathological events.

**RESULTS:**

Plasma glial fibrillary acidic protein (GFAP) and cerebrospinal fluid (CSF) soluble triggering receptor expressed on myeloid cells 2 (sTREM2) were increased in preclinical AD. Plasma GFAP showed an inverse bidirectional relationship with CSF amyloid beta (Aβ)42/40. CSF sTREM2 directly influenced CSF phosphorylated tau‐181 (p‐tau181) and neurogranin, and correlated with CSF S100 calcium‐binding protein beta (S100β). CSF chitinase‐3‐like protein 1 (YKL‐40) mediated the association between CSF p‐tau181 and total tau (t‐tau), whereas CSF S100β and neurofilament light showed mutual influence.

**DISCUSSION:**

Our findings suggest that microglial and astrocyte reactivity, measured through fluid biomarkers, occur early and impact the amyloid cascade on the preclinical Alzheimer´s continuum. Specifically, GFAP influences amyloid accumulation, sTREM2 promotes tau pathology, and YKL‐40 and S100β contribute to the progression of downstream neurodegenerative changes.

**Highlights:**

- Preclinical Alzheimer's disease (AD) showed increased levels of plasma glial fibrillary acidic protein (GFAP) and soluble triggering receptor expressed on myeloid cells 2 (sTREM2) compared to cerebrospinal fluid (CSF) in healthy subjects.
- Higher plasma GFAP levels was directly associated with lower CSF amyloid beta (Aβ)42/Aβ40.
- Higher CSF sTREM2 concentrations increased CSF phosphorylated tau‐181.
- Chitinase‐3‐like protein 1 (YKL‐40) mediated tau‐induced neurodegeneration.
- S100 calcium‐binding protein beta (S100β) was directly linked to higher neurofilament light (NfL) and showed a mutual relationship with sTREM2.

## 1 BACKGROUND

The contribution of neuroinflammation in Alzheimer´s disease (AD) pathogenesis has gained attention in recent years. Characterized by a mixed cellular response to brain pathological stimuli, primarily involving reactive microglia and astrocytes, neuroinflammation appears early in the course of AD—even before neurodegeneration and symptoms emerge.^1^ Many genetic loci associated with AD encode proteins related to glial function,^2^ supporting that neuroinflammation plays a key role in disease progression and represents an interesting therapeutic target.

Microglia are the resident macrophages and key components of the brain´s innate immunity. Triggering receptor expressed on myeloid cells 2 (TREM2) is expressed in microglia, and regulates their shift from a homeostatic to a disease‐associated state.^1^ The soluble form of TREM2 (sTREM2), detectable in cerebrospinal fluid (CSF) and plasma, is a well‐established biomarker of microglial reactivity in AD.^3^ However, studies show inconsistent results, with some reporting reduced CSF sTREM2 levels in mild cognitive impairment (MCI) or AD dementia compared to cognitively unimpaired (CU) individuals,^4^ whereas others find increased^3^, ^5^, ^6^, ^7^, ^8^ or unchanged levels.^9^, ^10^, ^11^, ^12^ It has been suggested that sTREM2 levels fluctuate across clinical AD stages, peaking before the onset of dementia.^3^, ^13^, ^14^ Research on autosomal dominant AD shows that CSF sTREM2 levels rise about 5 years before symptom onset,^8^ with similar findings observed in preclinical AD.^3^, ^7^, ^14^, ^15^ The role of TREM2 in AD remains controversial, and further investigation is needed.

Astrocytes respond to AD pathology by becoming reactive—undergoing molecular, functional, and morphological changes.^1^ Alterations in protein expression, such as glial fibrillary acidic protein (GFAP), chitinase‐3‐like protein 1 (YKL‐40), and S100 calcium‐binding protein β (S100β) are commonly observed.^16^, ^17^ Multiple astrocyte phenotypes likely exist, offering promising targets for biomarker and drug development.^18^ CSF GFAP and YKL‐40 are consistently elevated in patients with AD compared to controls.^14^, ^19^, ^20^ Of interest, CSF GFAP levels are strongly replicated in blood, and plasma GFAP consistently outperforms CSF GFAP as a biomarker of amyloid beta (Aβ) pathology in CU individuals.^19^, ^21^ Recent studies suggest that astrocytic changes appear early in AD, showing an upregulation of GFAP and YKL‐40 levels in Aβ‐positive CU individuals.^19^, ^22^, ^23^, ^24^, ^25^ In fact, astrogliosis may precede amyloid plaque formation, as confirmed by post‐mortem studies in MCI or preclinical AD^26^ and animal models.^26^ S100β is concentrated mainly in astrocytes. Elevated CSF S100β concentrations have been reported in AD and frontotemporal dementia compared to controls.^27^, ^28^ In AD animal models, overexpression of S100β increased brain Aβ deposits^29^ and Aβ42 levels in the hippocampus and frontal cortex.^30^ Conversely, inhibiting S100β reduced gliosis and amyloid pathology,^31^ whereas gene polymorphisms that increase its expression raised AD risk.^32^

The microglial and astrocyte response may influence critical processes of the amyloid cascade, including Aβ aggregation, tau pathology, neuronal injury, and cognitive decline. However, its specific impact on key early AD pathological events remains unclear. The glial response to AD is complex, and it remains unclear whether it plays a beneficial or detrimental role depending on factors such as disease stage or brain region, among others.^33^, ^34^ To date, most studies investigating glial biomarkers in preclinical AD focus specifically on either microglial or astrocytic biomarkers, but do not address both simultaneously, for a comprehensive and multidimensional approach.

**RESEARCH IN CONTEXT**

1. **Systematic review**: We conducted a literature review using PubMed and previously published reviews. Recent works on microglial and astrocytic experimental models, post‐mortem studies, neuroimaging, and fluid biomarker research are cited throughout the article.
2. **Interpretation**: Our findings support that microglial and astrocytic responses emerge early, during asymptomatic stages of Alzheimer's disease (AD). We observed that glial fibrillary acidic protein (GFAP)–related astrogliosis is triggered and also influences amyloid beta (Aβ) deposition in preclinical AD. soluble triggering receptor expressed on myeloid cells 2 (sTREM2)–related microglial response promotes tau pathology, whereas increased astrocyte reactivity, as measured by CSF YKL‐40 and CSF S100β, is associated with downstream neurodegenerative changes in the earliest stages of the Alzheimer´s continuum.
3. **Future directions**: Longitudinal studies across the entire Alzheimer's continuum, combining fluid and imaging biomarkers of microglial and astrocyte reactivity, are needed to clarify how different glial phenotypes impact AD progression at each disease stage and in specific brain regions.

Therefore, our aim was to assess changes in microglial (CSF sTREM2) and astroglia‐related (plasma and CSF GFAP, CSF YKL‐40, CSF S100β) biomarkers in the preclinical Alzheimer´s continuum in a well‐characterized cohort of CU individuals. Moreover, we sought to investigate the relationships between these glial biomarkers and early amyloid cascade events, including amyloid pathology (CSF Aβ42/40), tau pathology (CSF phosphorylated tau‐181 [p‐tau181]), synaptic dysfunction (CSF neurogranin), neuronal damage (CSF total tau [t‐tau] and neurofilament light [NfL]), and cognitive performance. We aimed to model the sequence of biomarker changes in preclinical AD. Using structural equation modeling, we sought to simultaneously analyze the complex interactions among these biomarkers, and investigate how glial cells influence these relationships in the earliest stages of AD.

## 2 METHODS

### 2.1 Study participants

The study was performed with volunteers from the “Valdecilla Cohort for the Study of Memory and Brain Aging” at the Memory Unit of Marqués de Valdecilla University Hospital (Santander, Spain), and it was approved by the hospital's ethics committee (Internal code: 2018.111). The project has been described in previous publications.^35^ In brief, it is a cohort designed to longitudinally study the preclinical phases of AD. The cohort consists of Caucasian CU individuals age 55 or older who have provided written informed consent for the collection and storage of biological samples. Exclusion criteria include the presence of any degree of cognitive impairment, defined by a Clinical Dementia Rating (CDR)^36^ >0; major psychiatric or systemic illness; sensory impairments that hinder the performance of cognitive tests; and any contraindication to performing complementary tests (e.g., claustrophobia or anticoagulation therapy).

At baseline, all participants undergo a lumbar puncture (LP)—to measure Aβ42, Aβ40, p‐tau181, and t‐tau—as well as a blood draw. In the first assessment, cranial magnetic resonance imaging (MRI), fluorodeoxyglucose–PET (FDG‐PET), and a comprehensive neuropsychological exam are also performed. Annual follow‐up assessments include a new blood draw and longitudinal neuropsychological evaluations.

Regarding the detailed participant selection process, 328 subjects responded to an open call in the media in our community. Of those 328 individuals, 60 participants are still awaiting their first evaluation. Among the remaining 268, a total of 26 were excluded due to unavailability (schedule conflicts and changes in residence), 13 were excluded because they were unable to undergo MRI or LP, 8 were excluded due to systemic or psychiatric conditions, 3 because of imaging findings (such as space‐occupying lesions or major stroke), and 7 were excluded because they had a score >0 on the CDR scale. In total, 211 subjects were included for analysis of CSF and plasma biomarkers (Figure S1).

### 2.2 Cognitive assessment

The neuropsychological assessment consists of a battery of tests that evaluate all cognitive domains. The details have been described in a previous work.^37^ The Mini‐Mental State Examination (MMSE)^38^ is used for global cognitive assessment and the global CDR score is used to establish the degree of dementia based on both functionality and cognition.

In this study a modified version of the Preclinical Alzheimer Cognitive Composite (PACC) score, known as the PACC5,^39^ was used. The PACC5 consists of the MMSE,^38^ the Logical Memory test from the Wechsler Memory Scale (total delayed recall),^40^ the Free and Cued Selective Reminding Test (Free + Total Recall),^41^ the Symbol Digit Modality Test,^42^ and the semantic fluency task (animals within 1 min).^43^ All raw test scores were standardized into *z*‐scores using the mean and standard deviation (SD) of CU participants as a reference, and then averaged into a composite score.

### 2.3 *APOE* status determination

We studied the apolipoprotein E (*APOE*) genotype using the TaqMan single nucleotide polymorphism genotyping assay (Applied Biosystems, Foster City, CA, United States). Subjects carrying ≥1 copy of the ε4 allele were classified as ε4+, whereas the remaining participants were placed in the ε4– group.

### 2.4 Sample collection and pre‐analysis

Our institution participates in the Alzheimer's Association Quality Control program and follows international recommendations for sample collection and storage.^44^, ^45^ CSF and plasma samples are collected on the same day in all participants, between 9:00 am and 10:00 am, with less than a 30‐min interval and with the subjects fasting. LPs are performed using a standard 22G needle, between the L3 and L5 spaces, with the patient in the lateral decubitus position. The CSF is collected into polypropylene tubes of 15 mL and then centrifuged at room temperature (2000 *g* for 10 min). The supernatant is aliquoted in 500 µL volumes into 1 mL tubes and frozen at −80°C until analysis in the immunology laboratory of our hospital.

Plasma samples are obtained according to the standardized operating procedures detailed in previous work.^46^ Blood is collected in 10 mL ethylenediaminetetraacetic acid (EDTA) tubes and kept cold until processed, which occurs within 3 h of collection. The samples are centrifuged (10 min at 1800 *g*). The resulting supernatant is stored in 500 µL aliquots in polypropylene tubes and frozen at −80°C until laboratory analysis.

### 2.5 CSF and plasma biomarkers

CSF Aβ40, Aβ42, p‐tau181, t‐tau, and NfL levels were measured in all participants using the automated immunoassay analyzer Lumipulse G600 II^47^ (Fujirebio Diagnostics, Malvern, PA, United States). The following kits were used: Lumipulse G β‐Amyloid 1‐40, Lumipulse G β‐Amyloid 1‐42, Lumipulse G p‐tau181, Lumipulse G t‐tau, and Lumipulse G NfL. Aβ42 and Aβ40 were used to calculate the Aβ42/Aβ40 ratio.

To establish the CSF cutoff points of Aβ42/Aβ42 ratio and p‐tau181, we applied an unbiased Gaussian mixture modeling approach^48^ based on a cohort of 578 subjects, which includes both CU and cognitively impaired subjects. Based on this model, we defined Aβ‐positive (A+) as CSF Aβ42/40 ratio <0.067, and tau‐positive (T+) as CSF p‐tau181 >55.0 pg/mL.

According to the biomarker profile, participants were divided into four groups: A−T−, A+T−, A+T+, and A−T+. Based on this classification, all individuals with abnormal Aβ values (A+T−, A+T+) were considered to be within the Alzheimer´s continuum. In addition, participants were further categorized into A+ and A− groups. A−T+ participants were excluded for the analyses based on amyloid status. According to the revised criteria for diagnosing and staging of AD of the Alzheimer´s Association,^49^ t‐tau was not considered as the “*N*” biomarker in terms of ATN classification due to its high correlation with p‐tau, particularly in preclinical stages. Nevertheless, given that t‐tau has been used widely as a marker of neurodegeneration in the existing literature, we retained it in this role—along with NfL—in subsequent analyses, including the structural equation modeling, with appropriate consideration of its interpretative limitations. Including both t‐tau and NfL in the same pathway analyses allows for a more comprehensive assessment of neurodegenerative processes, as the markers likely capture distinct yet complementary aspects of neuronal damage.

CSF levels of sTREM2, YKL40, S100β, and neurogranin were measured in all participants by using the Luminex 200 platform with a magnetic system (MILLIPLEX Human Neurodegenerative Disease) and analyzed with the Belysa Immunoassay Curve‐Fitting Software (both from Merck Life Science S.L.U., Madrid, Spain). The multiplex immunoassay was performed in 96‐well plates, which includes the following MILLIPLEX kits: HNS2MAG‐95K‐02 for sTREM2 and neurogranin, HCYP4MAG‐64K‐01 for YKL40, and HNDG4MAG‐36K‐01 for S100β.

In a subset of participants, plasma (*n* = 143) and CSF (*n* = 137) GFAP levels were measured using an in‐house ultrasensitive Single molecule array (Simoa) assay on an HD‐X Analyzer (Quanterix, Billerica, MA, USA) at the Clinical Neurochemistry Laboratory, University of Gothenburg, Sweden.

All CSF and plasma biomarker measurements described were performed using the same batch of reagents within their respective laboratories, in order to ensure analytical consistency across participants.

### 2.6 Statistical analysis

The Kolmogorov–Smirnoff test was used to assess the distribution of the variables. Non‐normally distributed CSF and plasma biomarkers were log10 transformed. According to these results, they have been described by mean and SD or median and interquartile range (IQR), as applicable.

To compare sample characteristics between groups we used analysis of variance (ANOVA) or Kruskal–Wallis test for quantitative variables and chi‐square test or Fisher's exact test for qualitative variables. We assessed the overall differences in glial biomarker levels between the biomarker‐defined groups using ANOVA. If statistically significant differences were found, a post hoc analysis was further performed to analyze the pairwise group differences, with the Bonferroni correction applied to adjust for multiple comparisons.

The Student´s *t*‐test was used to analyze differences in neuroinflammatory markers between A+ and A− subjects. In addition, general linear models (GLMs) were performed to assess differences in neuroinflammatory markers between groups, including age, sex, and *APOE* ε4 status as covariates. For each analysis, we considered the glial marker as the dependent variable, whereas the biomarker‐defined group (A−T−, A+T−, A+T−, A+T+) or the amyloid (A+ or A−) status served as independent variables, with age, sex, and *APOE* ε4 carriership as covariates. These comparisons were followed by Bonferroni corrected post hoc pairwise comparisons.

We used Pearson's correlation coefficient to analyze the raw correlations between CSF Aβ42/40 ratio, p‐tau181, t‐tau, NfL, and neurogranin levels with plasma GFAP, CSF GFAP, CSF sTREM2, CSF YKL‐40, and CSF S100β.

To investigate the association between glial and neuroinflammatory biomarkers and other markers of the AD cascade, we conducted linear regression analysis using neuroinflammatory biomarkers as predictors and core AD and neurodegeneration biomarkers as dependent variables. Age, sex, and *APOE* ε4 status were included as covariates in these models.

To explore relationships between core AD and glial biomarkers, we applied a Bayesian Network (BN) approach without imposing prior restrictions, enabling the visualization of conditional dependencies and probabilistic relationships without assuming causality. The Fast.iamb structure‐learning algorithm was used with 1000 bootstrap resampling iterations via the bnlearn package in R. Arcs with strength values >0.75, indicating that these connections appeared in at least 75% of the bootstrapped networks, were retained, providing moderate‐to‐high confidence in the identified relationships. Although the BN approach captured complex, multivariate dependencies and revealed data‐driven associations, it lacks *p*‐values and may be challenging to interpret for statistical inference. To address this, we used the BN‐derived relationships as a foundation for structural equation modeling, which allowed for enhanced interpretability, statistical significance testing, and the assessment of both direct and indirect effects.

To verify structural equation modeling assumptions, we examined multivariate normality using the MVN package in R and assessed linearity by inspecting scatterplot matrices for linearity. Model parameters were estimated using the Maximum Likelihood Robust (MLR) estimator within the lavaan package. Missing data were handled using the Maximum Likelihood (ML) method, assuming data were missing at random (MAR).

Our model was hypothesized based on both BN findings and the existing literature on the amyloid cascade in AD. We explored the potential mediating roles of reactive astrogliosis (elevated plasma GFAP, CSF YKL‐40, and S100β) and microglial reactivity (sTREM2) in AD biomarker progression, such as changes in amyloid, p‐tau, t‐tau, and neurodegeneration markers. To avoid redundancy, CSF GFAP was not included in this model in favor of plasma GFAP, which has demonstrated superior performance in detecting Aβ pathology in CU individuals.^19^, ^21^ A hierarchical model with six levels was constructed: (I) covariates age, sex, and *APOE* ε4 status; (II) CSF Aβ42/40 ratio as the initial pathological event; (III) astrocytic and microglial biomarkers as potential mediators; (IV) CSF p‐tau181 level, representing soluble tau pathology; (V) CSF t‐tau and NfL concentrations, indicating neurodegeneration, along with neurogranin for synaptic dysfunction; and (VI) global cognitive performance measured by the PACC5.

Relationships with clear directionality from the BN analysis were modeled as proposed, whereas those with undefined directionality were represented bidirectionally in the structural equation modeling framework. The model coefficients reflect both direct and indirect effects among biomarkers, quantifying complex multivariate relationships simultaneously in a single analysis. All biomarker data were standardized using *z*‐score scaling to ensure consistency across variables, facilitating direct comparison and reducing potential biases due to differences in variable scales. The 95% confidence intervals (CIs) for parameter estimates were calculated using MLR. Model fit was evaluated using multiple indices sensitive to overfitting, including the chi‐square to degrees of freedom ratio (χ^2^/df), the Root Mean Square Error of Approximation (RMSEA), the Standardized Root Mean Square Residual (SRMR), the Tucker‐Lewis Index (TLI), and the Comparative Fit Index (CFI). These indices strongly support the adequacy of the model, with values indicating good to excellent model fit (χ^2^/df = 1.5; RMSEA = 0.050, 90% CI: 0.016–0.076, p‐close = 0.477; SRMR = 0.038; TLI = 0.957, and CFI = 0.980).

For all the analyses, we applied a false discovery rate (FDR) multiple comparison correction following the Benjamini–Hochberg procedure. *p*‐Values ≤ 0.05 were considered as statistically significant. All statistical analyses were conducted using SPSS IBM version 25.0, R software version 4.4.1 (http://www.r‐project.org/) and Python (version 3.9.18). Figures were created with Python (version 3.9.18) and Canva (https://www.canva.com/).

## 3 RESULTS

### 3.1 Sample description

Our sample consisted of 211 CU volunteers, including 137 females (64.9%) and 74 males (35.1%). The median age was 64 years (IQR 60–69) and 63 participants (30.4%) were carriers of at least one *APOE* ε4 allele. The median score on the MMSE (0–30) was 29 (IQR 28–30). Table 1 summarizes the main demographic and clinical characteristics, CSF AD biomarker values, and the distribution of subjects across the AT groups.

**TABLE 1:** Participant characteristics.

Characteristic	Total (*N* = 211)
Female (%)	137 (64.9%)
Age, median (IQR)	64 (60–69)
*APOE* ε4 carrier, *n* (%)	63 (30.4%)
MMSE (0–30), median (IQR)	29 (28–30)
**CSF AD biomarkers**	
Aβ40, mean (SD), pg/mL	10868.4 (3215.3)
Aβ42, median (IQR), pg/mL	820.0 (578.0–1040.0)
Aβ42/40 ratio, median (IQR)	0.084 (0.064–0.093)
t‐tau, median (IQR), pg/mL	318.0 (241.0–400.0)
p‐tau181, median (IQR), pg/mL	37.6 (30.4–54.4)
**AT group, *n* (%)**	
A–T–	142 (67.3%)
A+T–	19 (9.0%)
A+T+	39 (18.5%)
A–T+	11 (5.2%)
**Physiological variables and comorbidities**	
eGFR, median (IQR), mL/min/1.73m^2^	93.7 (86.7–98.3)
Body mass index, mean (SD), kg/m^2^	26.3 (23.9–29.0)
Hypertension, *n* (%)	82 (38.5)
Diabetes mellitus, *n* (%)	17 (8.0)

*Note*: Sample characteristics.

Abbreviations: A, amyloid; *APOE*, apolipoprotein E; Aβ, amyloid beta; CSF, cerebrospinal fluid; eGFR, estimated glomerular filtration rate; IQR, interquartile range; MMSE, Mini‐Mental State Examination; *n*, number of subjects; p‐tau181, phosphorylated tau‐181; SD, standard deviation; T, tau; t‐tau, total tau.

### 3.2 Differences in microglial and astrocytic biomarkers across biomarker‐defined groups

Table 2 presents the main demographic and clinical characteristics of the sample, along with microglial and astrocyte‐related biomarker levels in each AT group.

**TABLE 2:** Differences in main characteristics and levels of glial markers across AT groups.

Characteristic / biomarker	A−T−	A+T−	A+T+	A−T+	*p*‐value
Female, *n* (%)	97 (68.3%)	16 (84.2%)	18 (47.2%)	6 (54.5%)	**0.016**
Age, median (IQR)	63 (59–68)	64 (62–70)	69 (64–72)	65 (61–75)	**<0.001** ^*^
BMI, median (IQR), kg/m^2^	26.4 (24.2–29.1)	29.0 (28.0–30.0)	29.0 (28.0–30.0)	29.0 (29.0–30.0)	**0.706** ^*^
MMSE (0–30), median (IQR)	29 (29–30)	29 (28–30)	29 (28–30)	29 (29–30)	**0.248** ^*^
Aβ40, mean (SD), pg/mL	10092.6 (2861.2)	10570.5 (2748.6)	12236.4 (2932.1)	16547.5 (1975.8)	**<0.001**
Aβ42, median (IQR), pg/mL	905.2 (280.5)	596.4 (145.7)	522.4 (169.8)	1390.2 (831.9)	**<0.001**
Aβ42/40 ratio, median (IQR)	0.089 (0.010)	0.057 (0.008)	0.043 (0.009)	0.084 (0.013)	**<0.001**
t‐tau, median (IQR), pg/mL	279.7 (81.0)	313.8 (71.5)	559.8 (215.3)	479.1 (75.3)	**<0.001**
p‐tau181, median (IQR), pg/mL	33.5 (9.4)	40.4 (9.2)	92.3 (4.4)	62.84 (7.3)	**<0.001**
CSF GFAP, median (IQR), pg/mL	484.50 (329.00–615.25)	490.00 (342.00–622.50)	678.00 (512.5–773.00)	520.00 (414.00–812.00)	**0.027**
Plasma GFAP, median (IQR), pg/mL	97.80 (72.70–120.00)	102.00 (87.45–159.00)	141.00 (107.05–243.50)	101.75 (78.43–135.00)	**<0.001**
CSF sTREM2, median (IQR), pg/mL	1464.52 (1026.47–1897.16)	1540.38 (1104.71–2100.02)	1779.81 (1147.07–2385.17)	1444.44 (1348.31–1974.14)	**0.025**
CSF YKL‐40, median (IQR), pg/mL	311.47 (225.19–434.54)	310.68 (143.33–473.18)	420.14 (265.80–561.82)	698.84 (223.58–1637.60)	**0.003**
CSF S100β, median (IQR), pg/mL	1122.13 (840.84–1452.59)	1100.08 (939.01–1385.84)	1320.31 (1031.39–1707.97)	1156.98 (874.60–1537.44)	0.571

*Note*: Data presented as the median (IQR) or as the *n* (%). Statistical significance was considered when *p*‐value ≤ 0.05 (ANOVA or Kruskal Wallis* test for quantitative variables; chi‐square test or Fisher's exact test for qualitative variables). *p*‐values ≤  0.05 are indicated in bold. *N* = 211. (Plasma GFAP, *n* = 143; CSF GFAP, *n* = 137.) Abbreviations: A, amyloid; ANOVA, analysis of variance; CSF, cerebrospinal fluid; GFAP, glial fibrillary acidic protein; IQR, interquartile range; MMSE, Mini‐Mental State Examination; *n*, number of subjects; S100β, S‐100 calcium‐binding protein beta; sTREM2, soluble triggering receptor expressed on myeloid cells 2; T, tau; YKL‐40, chitinase‐3‐like protein 1.

**FIGURE 1:**
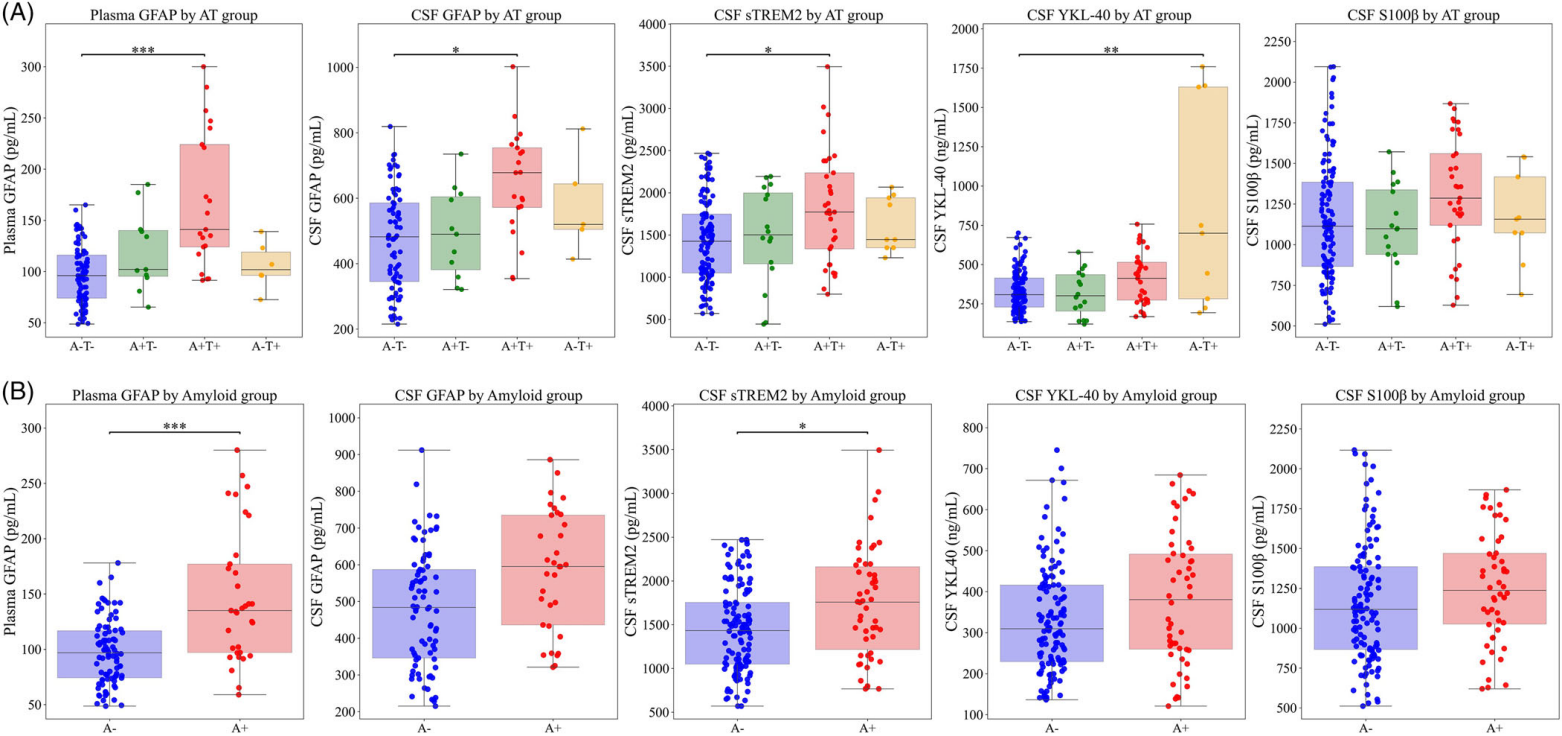
Differences in glial biomarkers according to AT group and amyloid status. The figure shows box‐and‐whisker plots of plasma and CSF GFAP, CSF sTREM2, CSF YKL‐40, and CSF S100β by AT group (1A) and amyloid status (1B). The x‐axis represents the different groups, whereas the y‐axis shows the concentrations of each glial biomarker, expressed in pg/mL, except for YKL‐40, which is in ng/mL. Outliers beyond the 95th and 5th percentiles were filtered prior to plotting. The boxes represent the IQR, with the upper boundary at Q3 and the lower boundary at Q1. The line inside the box indicates the median of the sample, and the whiskers show the most extreme data points within 1.5 times the IQR. Dots represent individual values. Significant differences are indicated by a horizontal line with one (*p* ≤ 0.05), two (*p* ≤ 0.01), or three (*p* ≤ 0.001) asterisks between the boxes. CSF, cerebrospinal fluid; GFAP, glial fibrillary acidic protein; sTREM2, soluble triggering receptor expressed on myeloid cells 2; YKL‐40, Chitinase‐3‐like protein 1; S100β, S100 calcium‐binding protein beta; A, amyloid; T, tau.

Significant differences were observed in plasma and CSF GFAP, CSF sTREM2, and CSF YKL‐40 across biomarker‐defined groups (Figure 1). Specifically, CSF and plasma GFAP and CSF sTREM2 were significantly higher in the A+T+ compared to the normal (A−T−) group. CSF YKL‐40 levels were elevated in the A−T+ compared to the A−T− group (Figure 1A).

After adjusting for age, sex, and *APOE* ε4, differences between AT groups in plasma GFAP (*F*(3, 136) = 6.26, *p*‐value: 0.001), CSF sTREM2 (*F*(3, 197) = 3.41, *p*‐value: 0.018), and CSF YKL‐40 levels (*F*(3, 198) = 5.29, *p*‐value: 0.002) remained significant (Table 3). No significant differences were found in CSF GFAP (*F*(3, 130) = 0.04, *p*‐value: 0.989) or S100β (*F*(3, 198) = 0.41, *p*‐value: 0.743) after adjustments for age, sex, and *APOE* ε4 carriership.

**TABLE 3:** Influence of AT group and amyloid status on plasma GFAP, CSF GFAP, CSF sTREM2, CSF YKL‐40, and S100β.

Group	Plasma GFAP	CSF GFAP	CSF sTREM2	CSF YKL‐40	CSF S100β
	*F* (df_M,_ df_R_)	*F* (df_M,_ df_R_)	*F* (df_M,_ df_R_)	*F* (df_M,_ df_R_)	*F* (df_M,_ df_R_)
AT	6.26^***^ (3, 136)	0.04 (3, 130)	3.41^*^ (3, 197)	5.29^**^ (3, 198)	0.41 (3, 198)
Amyloid	7.74^**^ (1, 130)	0.02 (1, 125)	8.50^**^ (1, 189)	0.10 (1, 189)	0.20 (1, 189)

*Note*: All models included age, sex, and *APOE* ε4 allele status as covariates. *N* = 211. (Plasma GFAP, *n* = 143; CSF GFAP, *n* = 137.)

Abbreviations: A, amyloid; GFAP, glial fibrillary filament protein; S100β, S‐100 protein β chain; sTREM2, soluble triggering receptor expressed on myeloid cells 2; T, tau;. CSF, cerebrospinal fluid; YKL‐40, chitinase‐3‐like protein 1.

* *p*‐value ≤  0.05.

** *p*‐value ≤  0.01.

*** *p*‐value ≤  0.001.

Post hoc analysis revealed that A+T+ subjects had significantly higher plasma GFAP levels than A+T− subjects (mean difference = 58.62 pg/mL, adjusted *p*‐value: 0.039; 95% CI: 115.38–1.86) and A−T− participants (mean difference = 64.76 pg/mL, adjusted *p*‐value: 0.001; 95% CI: 107.48–22.04).

CSF sTREM2 levels were higher in A+T+ compared to A−T− subjects (mean difference = 541.28 pg/mL, adjusted *p‐*value: 0.010; 95% CI 994.39–88.17). However, no differences in CSF sTREM2 levels were found between A+T+ and A+T− subjects (adjusted *p‐*value: 0.636) or between A+T+ and A−T+ subjects (adjusted *p*‐value: 0.618). Neither plasma GFAP or CSF sTREM2 differed between A+T− and A−T− individuals (adjusted *p‐*values: 1.000 and 0.875, respectively), or between A−T+ and A−T− participants (adjusted *p‐*values: 0.990 and 0.995, respectively).

A−T+ subjects had significantly higher CSF YKL40 levels compared to A−T− (mean difference = 422.54 ng/mL, adjusted *p*‐value: 0.001; 95% CI: 716.17–128.91), A+T+ (mean difference = 399.83 ng/mL, adjusted *p‐*value: 0.008; 95% CI 74.66–725.00) and A+T− subjects (mean difference = 483.05 ng/mL, adjusted *p*‐value: 0.002; 95% CI 128.72–837.38). No differences in CSF YKL40 were found between A+T+ or A+T− and A−T− subjects after adjusting for multiple comparisons (adjusted *p‐*values: 1.000 and 0.982, respectively).

### 3.3 Differences in microglial and astrocytic biomarkers by amyloid status

We conducted GLMs to assess differences in mean microglial and astrocytic marker levels between A+ and A− subjects, adjusting for age, sex, and *APOE* ε4 carriership.

A significant influence of the amyloid status was observed on plasma GFAP (F(1, 130) = 7.74, *p*‐value: 0.006) and CSF sTREM2 (F(1, 189) = 8.50, *p*‐value: 0.004), independent of age, sex, and *APOE* ε4 carriership, so a group analysis was performed. Plasma GFAP (142.84 pg/mL vs 104.41 pg/mL; mean difference: 38.43, *p‐*value < 0.0001, 95% CI: 11.10–65.76) and CSF sTREM2 (1855.32 pg/mL vs 1429.52 pg/mL; mean difference = 425.80, *p‐*value: 0.03, 95% CI 137.71–713.88) were significantly higher in A+ subjects compared to A− individuals (Figure 1B, Table 3). In contrast, amyloid status did not significantly influence CSF GFAP (F(1, 125) = 0.02, *p*‐value: 0.881), CSF YKL‐40 (F(1, 189) = 0.10, *p‐*value: 0.759), or CSF S100β levels (F(1, 189) = 0.20, *p*‐value: 0.887).

### 3.4 Correlation between microglia and astrocyte biomarkers and CSF Aβ, tau, synaptic, and neurodegeneration biomarkers

**FIGURE 2:**
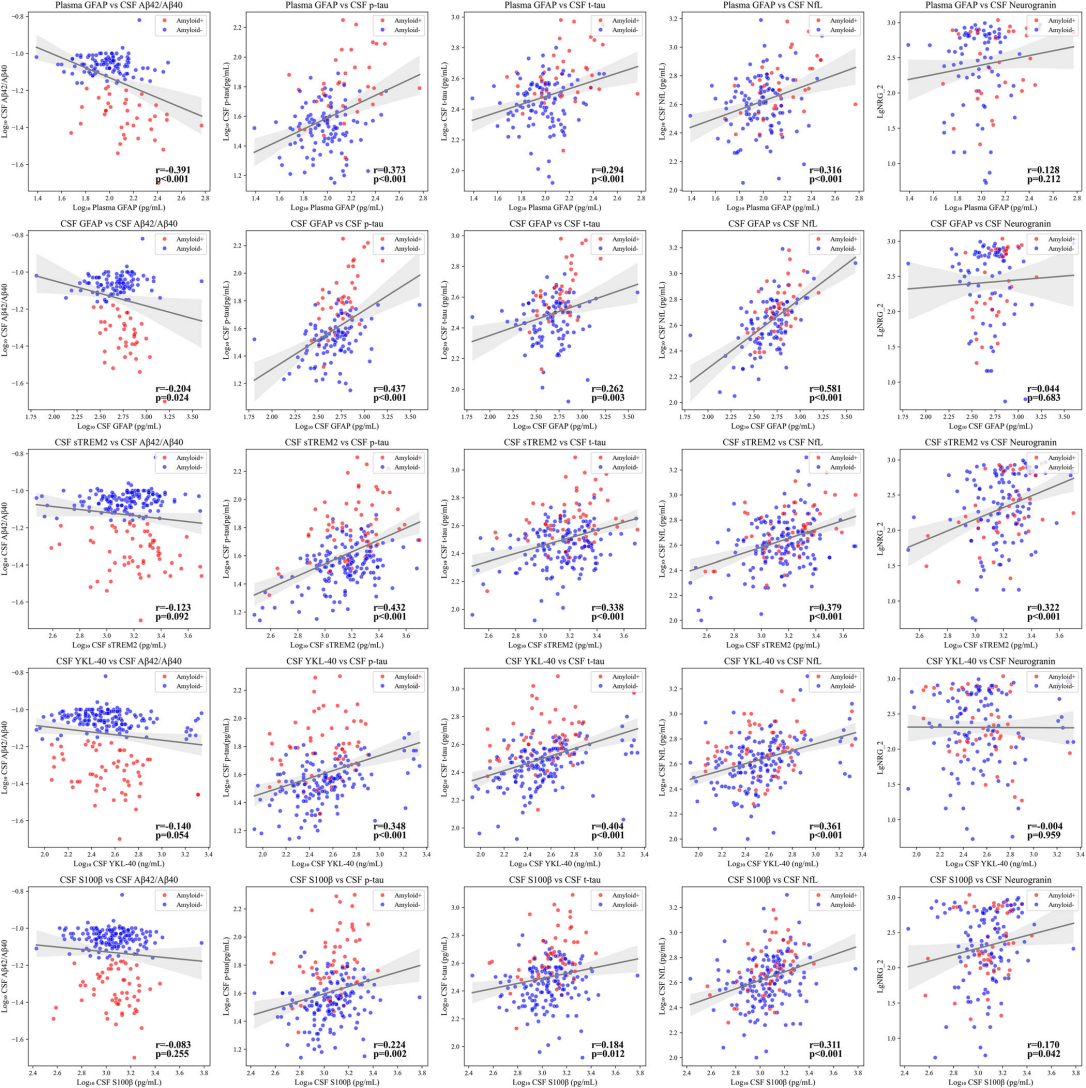
Correlation between each microglial and astrocytic biomarker and CSF pathological AD biomarkers. The plots show Pearson´s correlation coefficient between each glial marker and CSF Aβ42/Aβ40 ratio, p‐tau181, t‐tau, NfL, and neurogranin. The y‐axis represents the log10 values of CSF AD pathological biomarkers, whereas the x‐axis represents the log10 values of microglial and astrocytic biomarkers, expressed in pg/mL, except for YKL‐40, which is in ng/mL, and the amyloid ratio, which has no units. Dots represent paired values of both variables for each observation. The red line indicates the regression line, and the shaded area represents the 95% confidence interval. CSF, cerebrospinal fluid; AD, Alzheimer´s disease; p‐tau181, phosphorylated tau 181; t‐tau, total tau; NfL, neurofilament light; Aβ, amyloid beta; GFAP, glial fibrillary acidic protein; sTREM2, soluble triggering receptor expressed on myeloid cells 2; YKL‐40, Chitinase‐3‐like protein 1; S100β, S100 calcium binding protein beta.

We examined the correlations between plasma and CSF GFAP levels, CSF sTREM2, CSF YKL40, and CSF S100β with CSF biomarkers of the main pathogenic events described in AD (Figure 2; Table S1). All neuroinflammatory biomarkers showed statistically significant correlations with CSF p‐tau, t‐tau, and NfL levels after adjusting for multiple comparisons. In addition, CSF sTREM2 and S100β were significantly correlated with CSF neurogranin levels. Plasma and CSF GFAP levels were the only markers that showed significant correlations with the CSF Aβ42/40 ratio.

We performed linear regression analysis of the individual associations of glial and neuroinflammatory biomarkers with other markers of the AD cascade, considering age, sex and *APOE* ε4 carriership as covariates (Table 4). Higher plasma GFAP was associated with lower CSF Aβ42/40 ratio (β: −0.209, *p*‐value: 0.0002). Both plasma (β: 0.240, *p*‐value: 0.006) and CSF GFAP (β: 0.270, *p‐*value: 0.001) were positively associated with higher CSF p‐tau181 levels, and CSF GFAP was also associated with higher CSF NfL levels (β: 0.354, *p‐*value < 0.0001). Higher CSF sTREM2 levels were correlated with increased p‐tau181 (β: 0.384, *p*‐value < 0.0001), t‐tau (β: 0.246, *p*‐value < 0.0001), NfL (β: 0.284, *p‐*value < 0.0001), and neurogranin (β: 1.982, *p‐*value < 0.0001). CSF YKL‐40 was positively associated with *p*‐tau181 (β: 0.184, *p*‐value: 0.0004), t‐tau (β: 0.199, *p*‐value < 0.0001), and neurogranin (β: 0.631, *p*‐value: 0.04). Higher CSF S100β levels were associated with higher p‐tau181 (β: 0.190, *p*‐value: 0.008), NfL (β: 0.255, *p*‐value < 0.0001), and neurogranin (β: 1.226, *p*‐value: 0.03). No significant associations were found between any neuroinflammatory markers and cognitive performance on the PACC5 (Table 4).

**TABLE 4:** Linear regression analysis: Individual associations between plasma GFAP, CSF GFAP, CSF sTREM2, CSF YKL‐40, and S100β with AD cascade biomarkers

	Plasma GFAP	CSF GFAP	CSF sTREM2	CSF YKL‐40	CSF S100β
Aβ42/Aβ40	−0.209^***^	−0.044	−0.068	−0.036	−0.026
P‐tau181	0.240^**^	0.270^***^	0.384^****^	0.184^***^	0.190^**^
T‐tau	0.101	0.074	0.246^****^	0.199^****^	0.121
NfL	0.127	0.354^****^	0.284^****^	0.116	0.255^****^
Neurogranin	0.036	0.624	1.982^****^	0.631^*^	1.226^*^
PACC5	−0.394	0.042	0.097	0.290	−0.195

*Note*: Data are presented as unstandardized beta coefficients. All models included age, sex, and *APOE* ε4 allele status as covariates. *N* = 211. (Plasma GFAP, *n* = 143; CSF GFAP, *n* = 137).

Abbreviations: Aβ, amyloid beta; CSF, cerebrospinal fluid; GFAP, glial fibrillary acidic protein; NFL, neurofilament light; PACC5, Preclinical Alzheimer Cognitive Composite 5; p‐tau, phosphorylated tau; S100β, S‐100 calcium‐binding protein beta; sTREM2, soluble triggering receptor expressed on myeloid cells 2; YKL‐40, chitinase‐3‐like protein 1.

* *p*‐value ≤  0.05.

** *p*‐value ≤  0.01.

*** *p*‐value ≤  0.001.

**** *p*‐value ≤  0.0001.

### 3.5 Bayesian networks and structural equation model

The BN analysis replicated the theoretical AD cascade pathway and established several probabilistic relationships between neuroinflammatory markers and the pathological hallmarks of AD. These results are depicted in Figure S2. Briefly, the data supported the amyloid cascade model, with CSF Aβ42/40 associated with CSF p‐tau181, which was subsequently linked to CSF t‐tau and NfL. A strong association was observed between plasma GFAP and CSF Aβ, as well as between CSF sTREM2 and both CSF p‐tau181 and CSF neurogranin. In addition, CSF YKL40 was related to CSF t‐tau, and CSF S100β to CSF sTREM2. These findings, together with the existing literature, informed the hypothesized causal pathways that were later tested using structural equation modeling.

**FIGURE 3:**
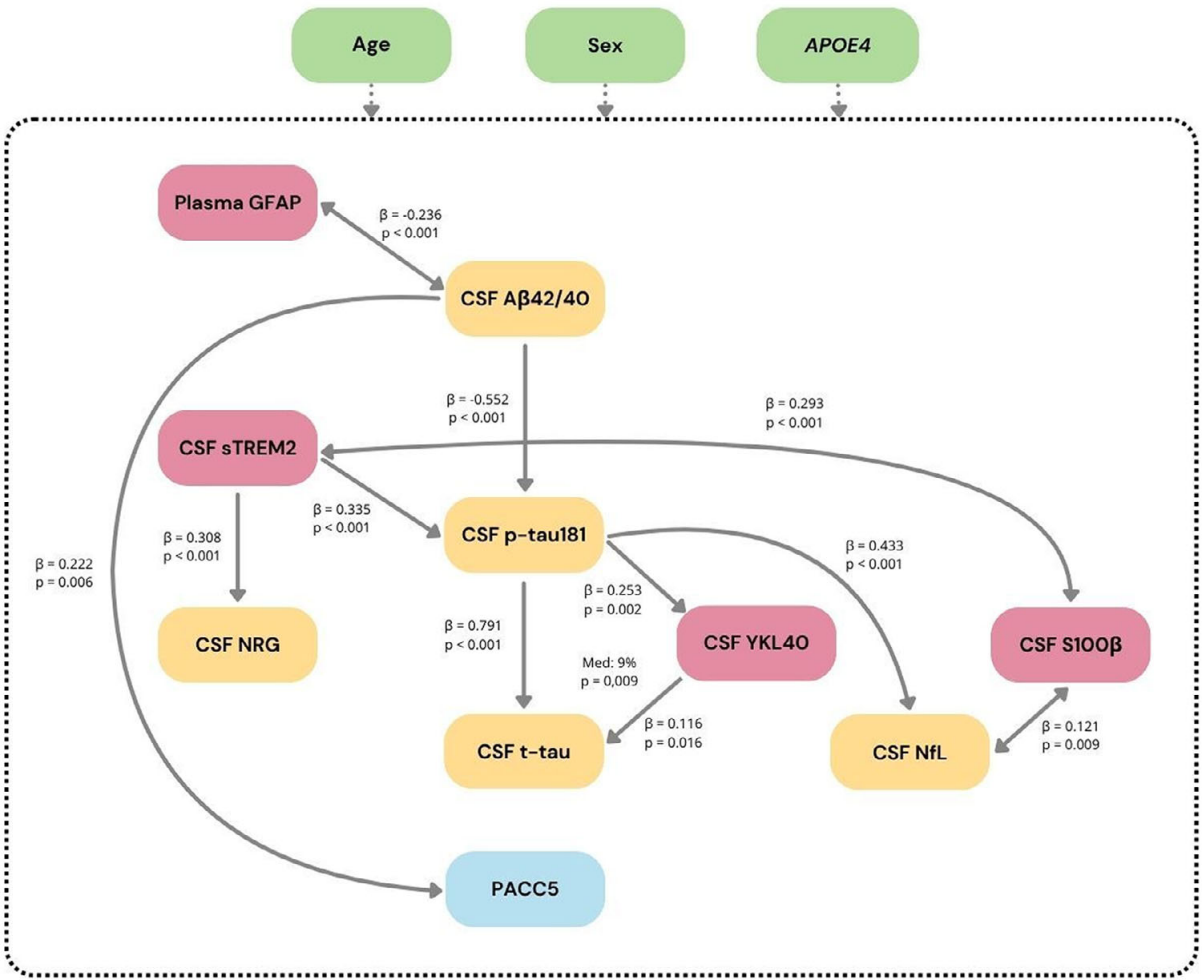
Structural equation model. Path analysis showing the impact of plasma GFAP, CSF TREM2, CSF YKL‐40, and S100β on amyloid, tau, synaptic dysfunction, neurodegeneration, and cognition. Cognition was measured by the PACC5 score. Arrows show the direct effects of significant associations at *p* ≤ 0.05 FDR‐corrected between all biomarker relationships (*z*‐score) from the structural equation model. The β estimates represent the unique contribution of a specific variable to the change in a dependent variable after controlling for the effects of age, sex, and *APOE* status and the rest of variables specified in each level of the model. CSF, cerebrospinal fluid; GFAP, glial fibrillary acidic protein; sTREM2, soluble triggering receptor expressed on myeloid cells 2; NRG, neurogranin; YKL‐40, chitinase‐3‐like protein 1; S100β, S100 calcium‐binding protein beta; PACC5, Preclinical Alzheimer Cognitive Composite 5.

The results of our structural equation modeling analysis are shown in Figure 3, where all significant direct associations between biomarkers are indicated with arrows. Table 5 displays all associations (both significant and non‐significant) along with their corresponding estimates and 95% CIs.

**TABLE 5 alz70502-tbl-0005:** Structural equation model coefficients showing all direct (directional and bidirectional relationships) and indirect effects of the path model.

Biomarker	β (95% CI)	Adjusted‐*p*‐value
CSF Aβ42/40		
Age	−0.044 (−0.063 to −0.024)	<0.001
*APOE* ε4 carrier	−0.892 (−1.175 to −0.609)	<0.001
Sex, female	0.192 (−0.07 to 0.455)	0.213
**Correlation with plasma GFAP**	−**0.236 (**−**0.362 to** −**0.11)**	<0.001
Plasma GFAP		
Age	0.082 (0.059 to 0.105)	<0.001
*APOE* ε4 carrier	0.188 (−0.115 to 0.492)	0.286
Sex, female	0.368 (0.083 to 0.653)	0.019
**Correlation with CSF Aβ42/40**	−**0.236 (**−**0.362 to** −**0.11)**	<0.001
CSF sTREM2		
Age	0.025 (0.005 to 0.044)	0.021
*APOE* ε4 carrier	−0.158 (−0.447 to 0.132)	0.347
Sex, female	−0.101 (−0.376 to 0.174)	0.546
**Correlation with CSF S100β**	**0.293 (0.155 to 0.432)**	<0.001
CSF p‐tau181		
Age	0.028 (0.013 to 0.043)	<0.001
*APOE* ε4 carrier	0.017 (−0.237 to 0.271)	0.925
Sex, female	−0.23 (−0.413 to −0.046)	0.022
**CSF Aβ42/40**	**−0.552 (−0.667 to −0.437)**	<0.001
**CSF sTREM2**	**0.335 (0.243 to 0.426)**	<0.001
CSF YKL40		
Age	0.048 (0.025 to 0.071)	<0.001
*APOE* ε4 carrier	−0.332 (−0.603 to −0.06)	0.026
Sex, female	−0.054 (−0.328 to 0.22)	0.761
**CSF p‐tau181**	**0.253 (0.104 to 0.402)**	0.002
CSF t‐tau		
Age	0.006 (−0.006 to 0.019)	0.395
*APOE* ε4 carrier	0.061 (−0.115 to 0.237)	0.560
Sex, female	0.044 (−0.099 to 0.186)	0.606
**CSF p‐tau181**	**0.791 (0.687 to 0.894)**	<0.001
**Mediated by CSF YKL40**	**0.091 (0.027 to 0.156)**	0.009
**CSF YKL40**	**0.116 (0.029 to 0.202)**	0.016
CSF S100β		
Age	0.026 (0.004 to 0.048)	0.029
*APOE* ε4 carrier	0.015 (−0.29 to 0.32)	0.925
Sex, female	−0.016 (−0.299 to 0.268)	0.925
**Correlation with CSF NfL**	**0.121 (0.036 to 0.207)**	0.009
**Correlation with CSF sTREM2**	**0.293 (0.155 to 0.432)**	<0.001
CSF NfL		
Age	0.053 (0.035 to 0.071)	<0.001
*APOE* ε4 carrier	−0.148 (−0.354 to 0.057)	0.216
Sex, female	0.494 (0.703 to 0.285)	<0.001
**CSF p‐tau181**	**0.433 (0.317 to 0.548)**	<0.001
**Correlation with CSF S100β**	**0.121 (0.036 to 0.207)**	0.009
CSF neurogranin		
Age	−0.002 (−0.024 to 0.02)	0.920
*APOE* ε4 carrier	0.207 (−0.056 to 0.469)	0.179
Sex, female	0.156 (−0.123 to 0.434)	0.340
**CSF sTREM2**	**0.308 (0.191 to 0.426)**	<0.001
PACC5		
Age	−0.069 (−0.091 to −0.048)	<0.001
*APOE* ε4 carrier	0.199 (−0.088 to 0.487)	0.234
Sex, female	0.151 (−0.09 to 0.393)	0.286
**CSF Aβ42/40**	**0.222 (0.078 to 0.366)**	0.006

*Note*: Structural equation model showing standardized coefficients with 95% confidence intervals calculated using the Maximum Likelihood Robust estimator. Model shown in Figure 4. *N* = 211 (Plasma GFAP, *n* = 143). *p*‐values adjusted for multiple comparisons using false discovery rate were considered significant at *p*‐value ≤ 0.05.

Abbreviations: *APOE*, apolipoprotein E; Aβ, amyloid beta; CI, confidence interval; CSF, cerebrospinal fluid; GFAP, glial fibrillary acidic protein; NfL, neurofilament light; PACC5, Preclinical Alzheimer Cognitive Composite 5; p‐tau, phosphorylated tau; S100β, S‐100 calcium‐binding protein beta; sTREM2, soluble triggering receptor expressed on myeloid cells 2; t‐tau: total tau; YKL‐40, chitinase‐3‐like protein 1.

We observed that older age (β = −0.044; 95% CI: −0.063 to −0.024) and particularly *APOE* ε4 carriership (β = −0.892; 95% CI: −1.175 to −0.609) showed a direct significant association with lower CSF Aβ42/40 ratio. Moreover, older age (β = 0.082; 95% CI: 0.059–0.105), female sex (β = 0.368; 95% CI: 0.083–0.653), and early Aβ pathology, as indicated by lower CSF Aβ42/40 ratio (β = −0.236; 95% CI: −0.362 to −0.11), were directly related to increased plasma GFAP. The inverse relationship between CSF Aβ42/40 ratio and plasma GFAP was bidirectional, so the estimates of the covariance are shown.

Furthermore, older age (β = 0.028; 95% CI: 0.014–0.043) and lower CSF Aβ42/40 ratio (β = −0.552; 95% CI: 0.668–0.437), showed a direct significant association with higher CSF p‐tau181 level. In addition, higher CSF sTREM2 showed a direct significant effect on elevated p‐tau181 (β = 0.355; 95% CI: 0.244–0.427). Increased CSF sTREM2 was also directly associated with elevated CSF neurogranin (β = 0.308; 95% CI: 0.191–0.426) and showed a bidirectional relationship with S100β (β = 0.293; 95% CI: 0.155–0.432).

In turn, higher p‐tau 181 showed a direct significant effect on increased CSF t‐tau (β = 0.823; 95% CI: 0.728–0.918) and CSF NfL levels (β = 0.432; 95% CI:  0.316–0.547). NfL levels were also influenced by older age (β = 0.053; 95% CI: 0.035–0.071) and male sex (β = −0.494; 95% CI: −0.703 to −0.285). Higher CSF YKL‐40 was significantly associated with older age (β = 0.048; 95% CI: 0.025–0.071), *APOE* ε4 (−) genotype (β = −0.332; 95% CI: −0.603 to −0.06), and higher CSF p‐tau (β = 0.253; 95% CI: −0.104 to 0.402). Besides, CSF YKL‐40 showed a significant effect on CSF t‐tau (β = 0.116; 95% CI: 0.029–0.202). CSF S100β and CSF NfL also showed a mutual influence (β = 0.122; 95% CI: 0.036–0.207). Finally, older age (β = −0.069; 95% CI: −0.091 to −0.048) and lower CSF Aβ42/40 ratio (β = 0.222; 95% CI: 0.078–0.366) were the only variables directly associated with worse cognitive performance, as measured by the PACC5.

### 3.6 Mediation effects

We observed that part of the relationship between CSF p‐tau181 and t‐tau could be explained by CSF YKL‐40 (Figure 3), with a proportion mediated of 9% (β = 0.09; 95% CI: 0.027–0.156). Despite the direct effect of sTREM2 on p‐tau181, no direct association between CSF Aβ42/40 and sTREM2 was observed. Thus, no CSF Aβ42/40–induced mediation by CSF sTREM2 on other biomarkers in the cascade was present. Similarly, plasma GFAP showed no direct relationship with CSF p‐tau181, and therefore no mediating effect of GFAP on the relationship between CSF Aβ42/40 and CSF p‐tau181 was found. Likewise, S100β did not show a direct association with CSF p‐tau181, and consequently, no induced mediation by S100β on the effect of CSF p‐tau181 on NfL was found.

## 4 DISCUSSION

In this cross‐sectional biomarker study, we assessed the correlates of early microglial and astrocytic responses in preclinical AD and their effects on downstream changes in the amyloid cascade, including soluble CSF p‐tau181, neurogranin, t‐tau, and NfL. To our knowledge, this study represents the first preclinical investigation in humans to simultaneously examine the interactions of both microglial and various astrocytic fluid biomarkers with AD pathology biomarkers, providing new insights into the complex interplay of glial cells in AD.[Table alz70502-tbl-0005]

According to previous studies,^23^, ^25^ we observed higher plasma GFAP and CSF sTREM2 in preclinical AD compared to normal subjects. Moreover, we observed elevated CSF YKL‐40 concentrations in A−T+ subjects but not in those within the Alzheimer´s continuum, suggesting that YKL‐40 may be involved in a non–amyloid‐related pathway. Although consistent with previous reports showing elevated CSF YKL‐40 in CU A+T+ and A−T+, but not in A+T− individuals^22^, ^25^, the limit size of our A‐T+ group warrants cautious interpretation.

Plasma GFAP was the only glial marker that correlated with Aβ. Accordingly, previous studies have demonstrated a strong relationship between GFAP and Aβ.^19^, ^21^, ^25^, ^50^ CSF sTREM2 was not associated with Aβ but did correlate with p‐tau181. These results align with previous work,^14^, ^51^ which also found no association between CSF Aβ and sTREM2. Both plasma and CSF GFAP, along with CSF sTREM2, YKL‐40, and S100β correlated, with downstream markers of synaptic dysfunction and/or neurodegeneration, suggesting a less specific involvement of sustained glial response in these pathological processes.

Our structural equation modeling analysis supported that plasma GFAP, CSF sTREM2, CSF YKL‐40, and CSF S100β significantly influence key events in the AD cascade at preclinical stages. Notably, we observed an inverse bidirectional relationship between CSF Aβ42/40 and plasma GFAP. Decreased CSF Aβ42/40 triggered an upregulation of plasma GFAP and, conversely, plasma GFAP decreased the CSF Aβ42/40. This mutual relationship suggests a close, reciprocal link between early amyloid deposition and GFAP‐related astrogliosis, reinforcing the notion of a tightly coordinated interaction between glial responses and amyloid pathology in the early stages of AD. In contrast, we found no association between plasma GFAP and tau pathology. Our results are consistent with a recent work that reported an influence of plasma GFAP on Aβ insoluble aggregates, but not on soluble tau, in CU subjects.^52^ Similarly, several studies observed that plasma GFAP correlated with amyloid‐PET, even in CU subjects.^19^, ^21^, ^23^ However, a recent work by Sánchez‐Juan et al. demonstrated an association between serum GFAP and post‐mortem tau pathology in advanced AD dementia.^53^ These findings suggest that GFAP‐related astrogliosis may be more directly involved with amyloid than tau pathology, at least in preclinical AD, supporting GFAP as a biomarker for early amyloid deposition. In late phases of AD, when amyloid has reached a plateau, GFAP could be more associated with tau and serve as a biomarker for disease monitoring.

In contrast to GFAP, the astrocytic biomarkers YKL‐40 and S100β were not associated with Aβ. Of interest, YKL‐40 and S100β release into the CSF occurred apparently later in the AD pathological cascade and was related with tau‐induced axonal damage. CSF YKL‐40 was associated with p‐tau181 and t‐tau, and partially mediated the relation between tau pathology and neuronal injury, as shown by Pelkmans et al.^52^ Although the role of t‐tau as a neurodegeneration marker is debated due to its correlation with p‐tau, our results suggest that YKL‐40 exerts an independent effect on t‐tau and mediates its association with p‐tau, supporting a contribution of YKL‐40–related astrogliosis to tau‐induced neuronal injury.

Consistent with our findings, in vivo studies have shown that CSF YKL‐40 correlates with tau pathophysiology in preclinical stages.^20^, ^25^, ^33^ Growing evidence demonstrates a positive association of CSF YKL‐40 with markers of neuronal injury, including cortical atrophy, CSF t‐tau, and NfL in early AD stages.^16^, ^20^, ^24^ These findings suggest that CSF YKL‐40 is closely tied to tau pathology and neuronal damage, highlighting the potential role of reactive astrocytes in impairing neuronal function.

CSF S100β was associated with axonal damage, as measured by CSF NfL. Prior studies reported a correlation between CSF S100β and neuronal degeneration markers, such as brain cortical atrophy, in AD.^27^ Experimental models demonstrated that S100β overexpression causes neurotoxicity, reduces neurogenesis, and increases tau phosphorylation.^54^ In addition to these findings, we suggest that in vivo CSF S100β may have influence on CSF NfL, as a marker of neuronal injury, in AD early stages. Of interest, we also observed a bidirectional relationship between CSF S100β and sTREM2, which may reflect the cross‐talk between astrocytes and microglia.^55^

Our SEM analysis supports that sTREM2‐related microglial response is an early event of the amyloid cascade in preclinical AD, tightly associated with p‐tau181 increases. This aligns with a recent post‐mortem study in which reactive microglia partially mediates the relationship between Aβ and tau.^56^ Likewise, another biomarker study in patients with MCI and AD dementia found that sTREM2 levels were higher in the T+ groups compared to T− groups, regardless of amyloid or cognitive status.^57^ Similarly, evidence from experimental models and PET studies supports that reactive microglia promote tau phosphorylation^58^, ^59^ and spread of tau deposits in AD.^60^ CSF sTREM2 also influences synaptic dysfunction, consistent with a recent study^61^ showing that sTREM2 mediates synaptic loss independently before tau accumulation becomes apparent. Conversely, other studies report protective effects of sTREM2 in reducing neurodegeneration and symptom progression in AD,^62^, ^63^ and TREM2 loss‐of‐function mutations are linked to higher AD risk^64^ and enhanced Aβ‐associated tau seeding in AD mice.^65^, ^66^

Consistent with previous studies, older age directly influenced YKL‐40 and plasma GFAP,^19^, ^22^, ^23^, ^52^, ^67^ whereas *APOE* ε4 status showed no relationship with glial biomarkers.^7^, ^23^, ^24^, ^52^ We did not find a contribution of sex on glial biomarkers, except for plasma GFAP, which exhibited higher concentrations in women. As described previously, CSF NfL was higher in men.^68^ Older age and lower CSF Aβ42/40 were the only variables directly related to cognitive performance. These results align with Pelkmans et al.,^52^ who found no direct relationship between GFAP and YKL‐40 and cognition in CU individuals, but differ from other studies reporting such an association.^69^, ^70^ The lack of relationship between glial markers and cognition may be due several factors: the limited test score variability due to the early disease stage, the cross‐sectional design, or the cognitive measure used, which is a composite of five tests but does not capture the full scope of neuropsychological assessment.

Our findings, together with those of the previous literature, suggest that reactive microglia and astrogliosis play a crucial role in the pathogenesis of AD through multiple pathways, detectable in the early asymptomatic stages of the disease. Glial responses likely occur at different points within the molecular AD cascade, including Aβ deposition, tau aggregation, synaptic dysfunction and neuronal degeneration. We hypothesize that astrocyte reactivity is triggered early by Aβ aggregates, with certain reactive astrocyte phenotypes expressing GFAP potentially contributing to Aβ accumulation. Aβ deposition and sustained microglial reactivity, with overexpression of sTREM2 and a continuous release of inflammatory mediators, may then promote tau hyperphosphorylation and neurofibrillary tangle formation. Misfolded Aβ and p‐tau perpetuate a persistent sterile type of immune reaction, which eventually impairs the function and structure of bystander neurons, leading to synaptic dysfunction and neurodegenerative changes. Certain astrocyte phenotypes expressing YKL‐40 and S100β may emerge later in the AD pathological cascade and contribute to downstream neurodegenerative changes, independently of amyloid pathology.

This study has some limitations. First, the data were collected cross‐sectionally, meaning we lack longitudinal data on neuroinflammatory markers. Second, our sample included only CU individuals, and thus the full clinical AD spectrum is not represented. Longitudinal studies spanning the entire AD continuum are necessary to explore how the relationship between neuroinflammatory and core AD biomarkers evolves over time. This is particularly important, as the immune response appears to follow a non‐linear trajectory, and the roles of reactive microglia and astrocytes in disease progression likely vary across disease stages.^71^, ^72^ Third, our path model offers a simplified view of the AD pathological cascade but does not fully capture the complexity of the disease. Fourth, we lack additional microglial biomarkers beyond sTREM2 to capture the full range of reactive microglial states. Despite these limitations, the study has notable strengths. We include a very well‐characterized cohort of CU subjects, and the use of the structural equation modeling approach enabled us to assess all direct and indirect effects on each variable within a single model, rather than analyzing each relationship separately. Moreover, to the best of our knowledge, this is the first preclinical AD study to simultaneously examine the interactions of both microglial and various astrocytic fluid biomarkers with AD pathology biomarkers. This integrated approach is crucial in understanding Alzheimer's as a multifactorial disease, in which a complex cascade of interconnected events ultimately contributes to its progression.

In conclusion, we provide compelling evidence that microglial and astrocyte biomarkers increase early on the AD continuum and have a significant influence on key pathogenic events at this stage. These results reinforce the idea that reactive microglia and astrocytes, in response to AD pathology, are crucial contributors to the progression of downstream neurodegenerative changes.

## CONFLICT OF INTEREST STATEMENT

K.B. has served on scientific advisory boards and/or as a consultant for Abbvie, AC Immune, ALZPath, AriBio, Beckman Coulter, BioArctic, Biogen, Eisai, Lilly, Neurimmune, Ono Pharma, Prothena, Roche Diagnostics, Sanofi, and Siemens Healthineers; has served on data‐monitoring committees for Julius Clinical and Novartis; has given lectures, produced educational materials, and participated in educational programs for Biogen, Eisai, and Roche Diagnostics; and is a co‐founder of Brain Biomarker Solutions in Gothenburg AB (BBS), which is a part of the GU Ventures Incubator Program, outside the work presented in this article. H.Z. has served on scientific advisory boards and/or as a consultant for Abbvie, Acumen, Alector, Alzinova, Alzinova, ALZPath, Amylyx, Annexon, Apellis, Artery Therapeutics, AZTherapies, Cognito Therapeutics, CogRx, Denali, Eisai, Enigma, LabCorp, Merck Sharp & Dohme, Merry Life, Nervgen, Novo Nordisk, Optoceutics, Passage Bio, Pinteon Therapeutics, Prothena, Quanterix, Red Abbey Labs, reMYND, Roche, Samumed, ScandiBio Therapeutics AB, Siemens Healthineers, Triplet Therapeutics, and Wave; has given lectures in symposia sponsored by Alzecure, BioArctic, Biogen, Cellectricon, Fujirebio, LabCorp, Lilly, Novo Nordisk, Oy Medix Biochemica AB, Roche, and WebMD; and is a co‐founder of Brain Biomarker Solutions in Gothenburg AB (BBS), which is a part of the GU Ventures Incubator Program (outside the submitted work). The other authors report no conflicts of interest. The author disclosures are to be found in the Supplementary Information.

## CONSENT STATEMENT

All subjects provided signed informed consent to participate in the study.

## Supporting information



Supporting Information

Supporting Information

Supporting Information

Supporting Information
