# Supplementary material for: Early microglial and astrocyte reactivity in preclinical Alzheimer's disease
Source: Alzheimers Dement. 2025 Aug 1;21(8):e70502. doi: 10.1002/alz.70502 (PMC12314543; doi:10.1002/alz.70502)
Supplement: Supplementary file 1 — Supporting Information [file ALZ-21-e70502-s003.pptx]

## Slide 1
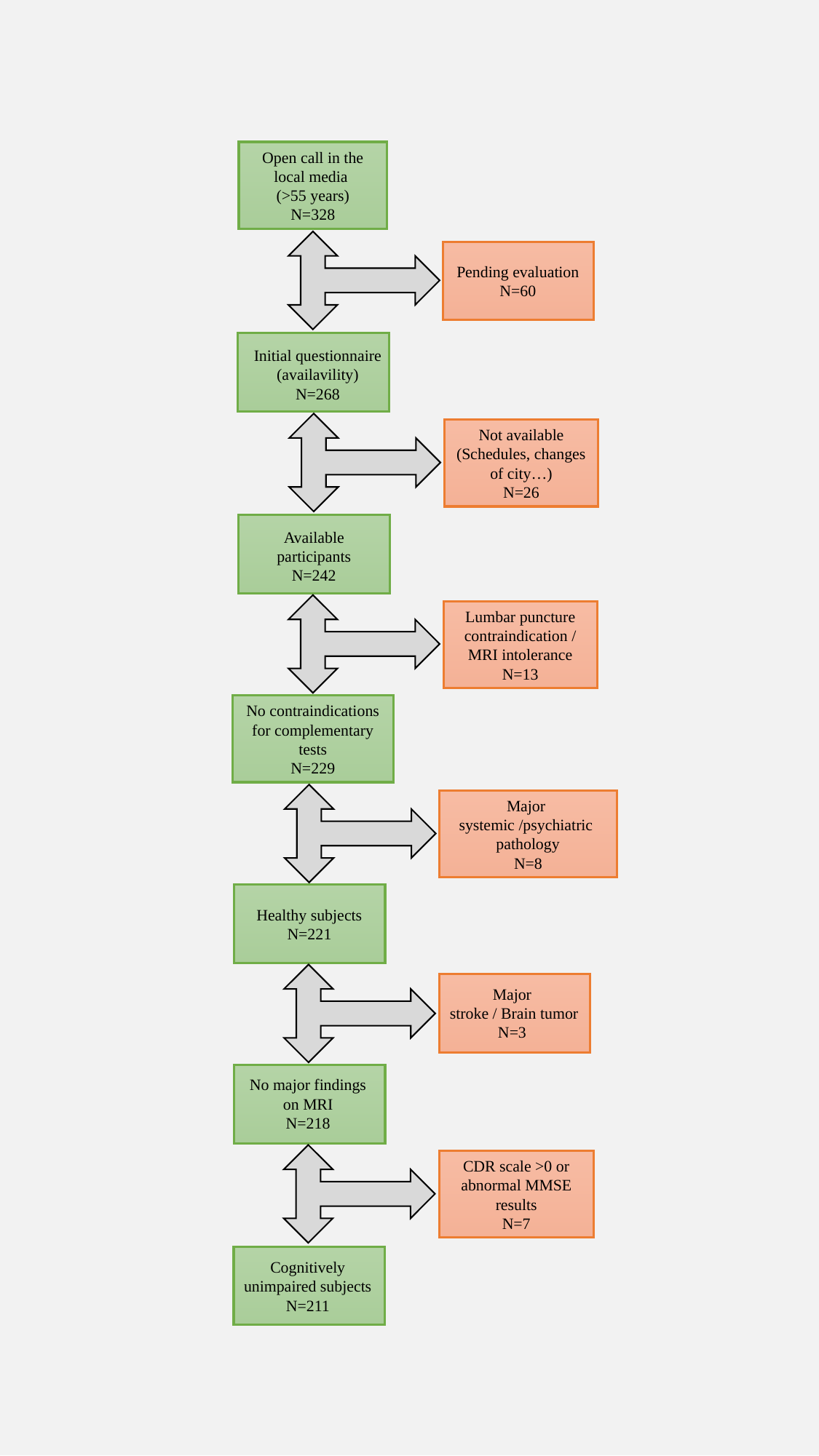

Open call in the local media
(>55 years)
N=328
Pending evaluation
N=60
Initial questionnaire (availavility)
N=268
Not available (Schedules, changes of city…)
N=26
Available participants
N=242
Lumbar puncture contraindication / MRI intolerance
N=13
No contraindications for complementary tests
N=229
Major
systemic /psychiatric
pathology
N=8
Healthy subjects
N=221
Major
 stroke / Brain tumor
N=3
No major findings on MRI
N=218
CDR scale >0 or abnormal MMSE results
N=7
Cognitively unimpaired subjects
N=211
