# Supplementary figures and images for: Early microglial and astrocyte reactivity in preclinical Alzheimer's disease
Source: Alzheimers Dement. 2025 Aug 1;21(8):e70502. doi: 10.1002/alz.70502 (PMC12314543; doi:10.1002/alz.70502)

## Slide 1
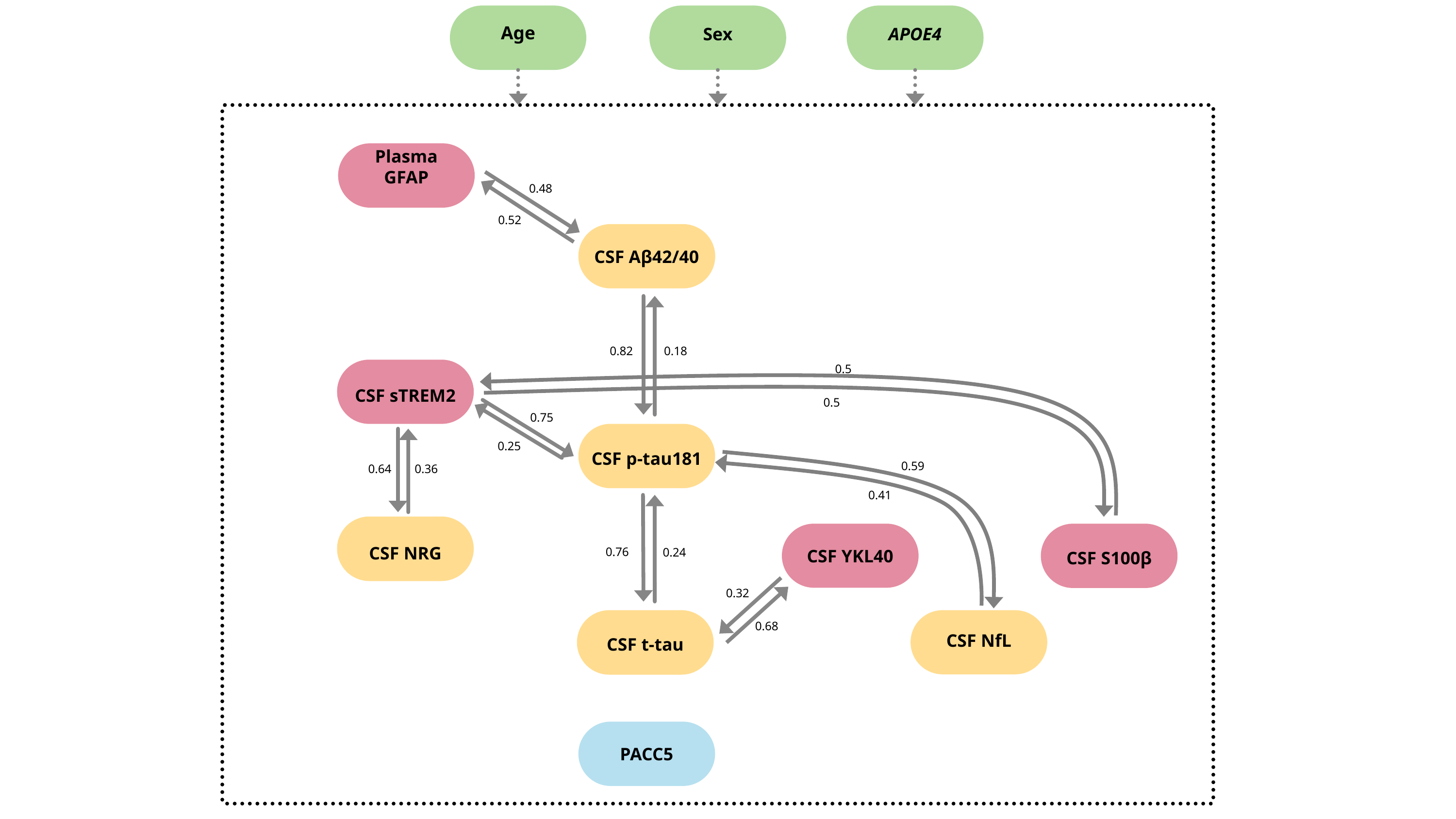

Age
Sex
APOE4
Plasma GFAP
0.48
0.52
CSF Aβ42/40
0.82
0.18
0.5
CSF sTREM2
0.5
0.75
CSF p-tau181
0.25
0.59
0.64
0.36
0.41
CSF S100β
CSF NRG
CSF YKL40
0.76
0.24
0.32
CSF t-tau
CSF NfL
0.68
PACC5

Supplement: Supplementary file 2 — Supporting Information [file ALZ-21-e70502-s001.pptx]
