# Supplementary material for: Early microglial and astrocyte reactivity in preclinical Alzheimer's disease
Source: Alzheimers Dement. 2025 Aug 1;21(8):e70502. doi: 10.1002/alz.70502 (PMC12314543; doi:10.1002/alz.70502)
Supplement: Supplementary file 3 — Supporting Information [file ALZ-21-e70502-s002.docx]

**Supplementary Table 1. Correlation between microglia and astrocyte biomarkers and AD CSF markers.**

|  | | | PLASMA AND CSF NEUROINFLAMATORY BIOMARKERS | | | | | | | | | |
| --- | --- | --- | --- | --- | --- | --- | --- | --- | --- | --- | --- | --- |
|  |  |  | **Plasma GFAP** | | **CSF GFAP** | | **CSF sTREM2** | | **CSF YKL40** | | **CSF S100β** | |
|  |  |  | r | Adjusted-*P* value | r | Adjusted-*P* value | r | Adjusted-*P* value | r | Adjusted-*P* value | r | Adjusted-*P* value |
| CSF BIOMARKERS | **Aβ42/Aβ40 ratio** | Overall | -0.39 | **<0.001** | -0.20 | **0.024** | -0.12 | 0.092 | -0.14 | 0.054 | -0.08 | 0.255 |
|  |  | A- | -0.05 | 0.720 | 0.14 | 0.240 | 0.16 | 0.083 | -0.18 | 0.052 | -0.03 | 0.726 |
|  |  | A+ | -0.33 | 0.069 | -0.48 | **0.008** | -0.14 | 0.320 | -0.29 | **0.032** | -0.07 | 0.642 |
|  | **P-tau181** | Overall | 0.37 | **<0.001** | 0.44 | **<0.001** | 0.43 | **<0.001** | 0.35 | **<0.001** | 0.22 | **0.002** |
|  |  | A- | 0.11 | 0.329 | 0.36 | **0.001** | 0.43 | **<0.001** | 0.45 | **<0.001** | 0.19 | **0.036** |
|  |  | A+ | 0.38 | **0.032** | 0.63 | **0.001** | 0.46 | **0.002** | 0.34 | **0.023** | 0.32 | **0.032** |
|  | **T-tau** | Overall | 0.29 | **<0.001** | 0.26 | **0.003** | 0.34 | **<0.001** | 0.40 | **<0.001** | 0.18 | **0.012** |
|  |  | A- | 0.10 | 0.364 | 0.08 | 0.481 | 0.27 | **0.003** | 0.46 | **<0.001** | 0.10 | 0.274 |
|  |  | A+ | 0.27 | 0.126 | 0.59 | **0.001** | 0.38 | **0.012** | 0.39 | **0.010** | 0.36 | **0.016** |
|  | **NfL** | Overall | 0.32 | **<0.001** | 0.58 | **<0.001** | 0.38 | **<0.001** | 0.36 | **<0.001** | 0.31 | **<0.001** |
|  |  | A- | 0.17 | 0.132 | 0.57 | **<0.001** | 0.31 | **<0.001** | 0.41 | **<0.001** | 0.25 | **0.005** |
|  |  | A+ | 0.42 | **0.023** | 0.55 | **0.002** | 0.50 | **0.001** | 0.23 | 0.098 | 0.48 | **0.001** |
|  | **Neurogranin** | Overall | 0.13 | 0.212 | 0.04 | 0.683 | 0.32 | **<0.001** | 0.00 | 0.959 | 0.17 | **0.042** |
|  |  | A- | 0.06 | 0.629 | -0.08 | 0.615 | 0.24 | 0.056 | 0.07 | 0.615 | 0.14 | 0.316 |
|  |  | A+ | -0.28 | 0.126 | 0.49 | **0.01** | 0.53 | **0.001** | -0.23 | 0.126 | 0.25 | 0.126 |

Note: Correlations between neuroinflammatory biomarkers and other CSF AD markers using Pearson's correlation coefficient. Adjusted P-values based on False Discovery Rate (FDR) procedure are shown. Adjusted-P values ≤ 0.05 are indicated in bold. N = 211. (Plasma GFAP, n = 143; CSF GFAP, n = 137). Abbreviations: CSF, cerebrospinal fluid. Aβ, amyloid β. P-tau, phosphorylated tau. NfL: neurofilament light chain. GFAP, glial fibrillary filament protein. sTREM2, soluble triggering receptor expressed on myeloid cells 2. YKL-40, chitinase-3-like protein 1. S100β, S-100 protein β chain. A, amyloid status. r, Pearson’s correlation coefficient.
